# A Novel Cell-Penetrating Peptide–Vascular Endothelial Growth Factor Small Interfering Ribonucleic Acid Complex That Mediates the Inhibition of Angiogenesis by Human Umbilical Vein Endothelial Cells and in an Ex Vivo Mouse Aorta Ring Model

**DOI:** 10.34133/bmr.0120

**Published:** 2025-01-07

**Authors:** Minseo Kim, Sangkyu Park, Soyi Kim, Jeongmin Seo, Sangho Roh

**Affiliations:** ^1^ Cellular Reprogramming and Embryo Biotechnology Laboratory, Dental Research Institute, Seoul National University School of Dentistry, Seoul 08826, Republic of Korea.; ^2^ Biomedical Research Institute, NeoRegen Biotech Co., Ltd., Suwon, Gyeonggi 16614, Republic of Korea.

## Abstract

Angiogenesis is mediated by vascular endothelial growth factor (VEGF), a protein that plays a key role in wound healing, inflammatory diseases, cardiovascular processes, ocular diseases, and tumor growth. Indeed, modulation of angiogenesis represents a potential approach to treating cancer and, as such, therapeutic approaches targeting VEGF and its receptors have been widely investigated as part of the broader search for curative interventions. Equally, RNA interference is a powerful tool for treating diseases, but its application as a disease treatment has been limited in part because of a lack of efficient small interfering RNA (siRNA) delivery systems. The purpose of this study was to characterize an amphipathic cell-penetrating peptide, Ara27, and its potential as an effective delivery vehicle as a conjugate with VEGF siRNA (siVEGF). In our study, we demonstrate that exposure of human umbilical vein endothelial cells (HUVECs) with Ara27–siVEGF complexes did not lead to cytotoxicity and can lead to down-regulation of cellular levels of both VEGF mRNA and protein. Moreover, treatment with the Ara27–siVEGF complex attenuates the phosphorylation of VEGFR2, Akt, and ERK in HUVECs and inhibits their capacity for wound healing and tube formation, both of which characteristics reflective of angiogenesis. In addition, we performed an ex vivo study to find that treatment with the Ara27–siVEGF complex inhibits aorta ring sprouting. Furthermore, the complex did not induce immunotoxicity in THP-1 and RAW264.7 cells. Taken together, our studies demonstrate that an Ara27–siVEGF conjugate is efficient for knockdown of VEGF in HUVECs to inhibit angiogenesis, without marked cytotoxic and immunotoxic effects.

## Introduction

Angiogenesis is the process in which new blood vessels form through cellular expansion of the surrounding vascular network [1]. Angiogenesis is essential for growth and development and plays a key role in wound healing, inflammatory diseases, cardiovascular processes, ocular diseases, and, in particular, tumor growth and metastasis [2,3]. Angiogenesis is regulated by pro- and anti-angiogenic factors, and changes in this equilibrium can activate a molecular angiogenic signaling pathway, leading to pathological vessel formation [4]. Vascular endothelial growth factor (VEGF) is one of the major pro-angiogenic molecules that stimulate this pathway, stimulating endothelial cell proliferation, migration, and neovascularization through crosstalk with VEGF receptor-2 [5]. Because of these critical functions by these signaling factors, researchers have developed drugs to modulate angiogenesis by targeting VEGF and its receptors. Some of these drugs are in clinical use, including monoclonal antibodies, small-molecule tyrosine kinase inhibitors, and molecular inhibitors of signaling pathways [6]. However, because these drugs require administration at high doses to maintain their effectiveness, nonspecific binding, unexpected toxicities, and side effects have been found to be associated with their use [7–9]. Therefore, the development of novel anti-angiogenic agents with high efficacy and low toxicity is imperative to improve the targeted treatment of VEGF signaling in homeostasis, disease, and injury states.

RNA interference is emerging as a promising therapeutic approach to modulating gene expression by sequence-specific mRNA degradation [10]. Small interfering RNAs (siRNAs) have been explored as a potential gene therapy for the treatment of various classes of inherited and acquired diseases because of their high targeting specificity and low side-effects [11,12]. However, the efficient delivery of siRNA to sites of action and cells of interest remains a challenge owing to its pharmacological properties. Notably, the phosphate groups on the surface of siRNAs render them difficult to diffuse across cellular membranes because they are highly anionic [13]. Nevertheless, in the past several years, a variety of approaches have been explored to improve siRNA pharmacokinetics, cellular delivery, and intracellular trafficking [14,15]. Presently, viral vectors are highly restricted in their use as vehicles for siRNA delivery in clinical applications due to the concerns of their strong immunogenicity, high toxicity, and inflammatory reactions. On the other hand, nonviral carriers are emerging as promising therapeutic nucleic acid delivery tools because of their biocompatibility and physicochemical properties [16].

Cell-penetrating peptide (CPP) is defined as a short (~5 to 30 amino acids) peptide that has cell membrane protein transduction domains or membrane translocating sequences. They comprise cationic or amphipathic sequences that can cross the cell membranes [17]. Indeed, CPPs have been reported to be able to deliver various kinds of cargo into target cells, such as fluorophores, drugs, peptides and nucleic acids [18,19]. By interacting noncovalently with CPP, siRNA can be delivered by forming complexes with both the cationic and anionic parts of CPPs [20,21]. In the case of cationic CPPs, their charge can be masked when combined with nucleic acids, such that the resulting CPP–siRNA mixture is not efficiently taken up into cells [22]. In contrast, amphipathic CPPs mixed with siRNAs have been found to have greater cellular uptake, cytosolic localization, endosomal escape properties and gene silencing effects [23–25]. One such example, Ara27, an amphipathic CPP, is efficiently internalized even at low concentrations and short treatment conditions in various cell lines, without evidence of cytotoxicity [26]. A significant improvement in intracellular uptake was shown with Ara27 as compared to commonly used CPPs, such as Tat-protein transduction domain and membrane translocating sequence, without adverse effects on the viability of the cells [27].

In this study, we prepared Ara27–siVEGF complexes and characterized them through electrophoretic mobility shift assay and size analysis. We then treated human umbilical vein endothelial cells (HUVECs) with Ara27–siVEGF and observed that this treatment facilitated the delivery of siVEGF molecules into the cytosol without any evidence of cytotoxicity. Expression analyses revealed that both VEGF mRNA and protein expression were down-regulated in HUVECs following Ara27–siVEGF treatment. Furthermore, we conducted wound healing and tube formation assays with HUVECs, which demonstrated that treatment with Ara27–siVEGF influenced cellular behaviors in a manner associated with the inhibition of VEGF receptor-2 (VEGFR2), Akt, and extracellular signal-regulated kinase (ERK) signaling pathways. The effects of Ara27–siVEGF on angiogenesis were further supported by results from an ex vivo mouse aortic ring sprouting assay, which showed that treatment with Ara27–siVEGF suppressed sprouting. Notably, Ara27–siVEGF did not induce immunotoxicity in THP-1 and RAW264.7 cells, underscoring its safety profile.

## Materials and Methods

### Materials

Ara27, an amphipathic cell penetrating peptide (CPP), was synthesized by LifeTein (LifeTein LLC, NJ, USA) using PeptideSyn technology based on FMOC (9-fluorenylmethoxy carbonyl) chemistry. Ara27 was labeled with fluorescein isothiocyanate (FITC). The sequence of Ara27 is RNQRKTVRCFRCRQAGHWISDCRLKSK. The siRNA oligos used in this study were synthesized by Bioneer (Daejon, Korea). The sequences of the negative control siRNA (siNC) and VEGF siRNA (siVEGF) are shown in Table 1. The siVEGF sequence was previously reported [28,29]. Cyanine3 (Cy3)-labeled siVEGF was also synthesized by Bioneer. TransITx2 was purchased from Mirus Bio (WI, USA) for using as positive control on transfection. Axitinib was obtained from Selleckchem (TX, USA).

**Table 1.** Sequence information of the siRNAs used in this study

Name	Sequence (5′ to 3′)
si*VEGF*	Sense: AUG UGA AUG CAG ACC AAA GA TT
	Antisense: UUC UUG GUC UGC AUU CAC AU TT
si*NC*	Sense: UUC UCC GAA CGU GUC ACG U TT
	Antisense: ACG UGA CAC GUU CGG AGA A TT

### Peptide structure prediction

The NetSurfP web server (https://services.healthtech.dtu.dk/services/NetSurfP-2.0) was used to predict relative surface accessibility and secondary structure prediction of the residue in the Ara27 peptide [30]. The I-TASSER server (http://zhanglab.ccmb.med.umich.edu/I-TASSER) was also used to predict the structure of the Ara27 peptide [31].

### Preparation of the CPP–siVEGF complex

Preparations of siVEGF (final concentration, 50 nM) and Ara27 mixtures were prepared in different molar ratios (1:1, 1:5, 1:10, 1:20, and 1:30). The Ara27–siVEGF complexes for in vitro experiments were prepared in PBS containing 5% glucose and incubated at 37 °C for 30 min. TransITx2 (Mirus Bio) was used as positive control on transfection. In all experiments, 3 μl of TransITx2 was used per 1 ml of growth medium according to the manufacturer's instructions. The siRNA was mixed with TransITx2 in Opti-MEM at 10% by volume of the growth medium. The mixture was incubated for 20 min at room temperature and cells were treated dropwise.

### Electrophoretic mobility shift assay

Agarose gel electrophoresis was utilized to study siRNA binding affinity to Ara27. The CPP–siVEGF complexes were formed at different molar ratios (1:1, 1:5, 1:10, 1:20, and 1:30) with a constant amount of siRNA (100 pmol). After incubation for 30 min at 37 °C, the complexes mixed with a 6× loading dye were loaded into 2% (w/v) agarose gel dissolved in 100 ml of Tris-acetate-EDTA (TAE) buffer. The 2% agarose gel was electrophoresed at 70 V for 40 min in TAE buffer. Images of the electrophoretic mobility shifts of complexes separated within the gel were captured using an InGenius System (Syngene, Cambridge, UK).

### Characterization of CPP–siVEGF complexes

Ara27–siVEGF complexes (prepared as per above) were measured by dynamic light scattering (DLS; Zetasizer Ultra; Malvern Instruments, Worcestershire, UK) to measure mean size (*Z*-average) of the particle distribution and homogeneity (polydispersity index [PDI]).

### Cell culture

HUVECs were obtained from Promocell (Heidelberg, Germany). The HUVECs were cultured in Endothelial Cell Growth Medium (Promocell) and incubated at 37 °C in a humidified atmosphere with 5% CO_2_. HUVECs at passages 4 to 9 were used for all experiments. The human monocytic leukemia cell line THP-1 was purchased from the American Type Culture Collection (ATCC; Virginia, USA). THP-1 cells were cultured in Roswell Park Memorial Institute 1640 medium supplemented with 10% fetal bovine serum (FBS) and 1% penicillin/streptomycin at 37 °C in 5% CO _2_. THP1 cells were differentiated by phorbol 12-myristate 13-acetate (PMA) for 48 h. Murine RAW264.7 macrophages were obtained from ATCC. The cells were cultured in Dulbecco’s modified Eagle's medium (DMEM) supplemented with 10% FBS and 1% penicillin/streptomycin. Cells were cultured in an incubator at 37 °C in a humidified 5% CO_2_.

### Cell viability assay

A Cell Counting Kit-8 (CCK-8; Abbkine, Wuhan, China) was used, based on water-soluble tetrazolium salt (WST-8). HUVECs were seeded at a density of 4,000 cells/well on 96-well plates. After 24 h, the cells were treated under different conditions and incubated at 37 °C. After 24 h of treatment, media from each well were replaced with fresh media containing 10 μl of CCK-8 reagents and incubated at 37 °C for 1 h. The absorbance was measured at 452 nm using a Multiskan GO microplate spectrophotometer (Thermo Fisher Scientific, MA, USA).

### Lactate dehydrogenase assay

To measure cell death, cell culture supernatants were collected and lactate dehydrogenase (LDH) was analyzed using an LDH assay kit (DoGenBio, Seoul, Korea) according to the manufacturer's instructions. Optical density (OD) was measured at 450 nm using a Multiskan GO microplate spectrophotometer (Thermo Fisher Scientific). The LDH release was calculated as % = (OD of each sample/OD of lysis sample) × 100.

### Cellular internalization observed using confocal microscopy

HUVECs were seeded on gelatin-coated coverslips at an appropriate density. After 24 h of stabilization, the cells were treated with Ara27-FITC (1 μM), siVEGF-cy3 (50 nM), TransITx2 siVEGF-cy3 (50 nM), and 1:5, 1:10, 1:20, and 1:30 molar ratios of the Ara27–siVEGF complex. After 24-h incubation, immunofluorescence staining was conducted. Briefly, cells on coverslips were washed 3 times with heparin-containing PBS and fixed for 10 min with 4% paraformaldehyde solution. Next, the coverslips were rinsed thoroughly with PBS and mounted on slides using a mounting solution containing Hoechst 33342. Confocal microscopy was conducting using an LSM800 instrument (Zeiss, Munich, Germany). Ara27 was labeled with FITC at its C-terminal end and siVEGF was labeled with Cy3 at the 5′-end of its sense strand. Information on the fluorescence wavelengths is shown in Table S1.

### Evaluation of relative mRNA expression by RT-qPCR

A TaKaRa MiniBEST Universal RNA Extraction Kit (Takara, Tokyo, Japan) was used to isolate total RNA from cultured cells, followed by reverse transcription to complementary DNA. RT-qPCR reactions were performed using a 7500 real-time PCR system (Applied Biosystems, CA, USA). The ΔΔCT method was employed to calculate relative expression. The expression of target mRNA was adjusted to that of internal control glyceraldehyde 3-phosphate dehydrogenase (GAPDH) in the same sample. Each value represents the average of 3 independently performed runs with standard deviations. The details of genes tested in this study and their respective nucleotide primer sequences are shown in Table 2.

**Table 2.** Sequence information of the primers used in this study

Species	Gene	Primer sequence (5′ to 3′)
Human	VEGF	Forward: GCAGCTTGAGTTAAACGAACG
		Reverse: GGTTCCCGAAACCCTGAG
Human	IL-6	Forward: CAATGAGGAGACTTGCCTGG
		Reverse: GCACAGCTCTGGCTTGTTCC
Human	IL-1β	Forward: TGGCAATGAGGATGACTTGTTC
		Reverse: CTGTAGTGGTGGTCGGAGATT
Human	TNF-α	Forward: AACCTCCTCTCTGCCATCAA
		Reverse: GGAAGACCCCTCCCAGATAG
Human	GAPDH	Forward: TGGACTCCACGACGTACTCA
		Reverse: ACATGTTCCAATATGATTCC
Mouse	IL-6	Forward: GAGGATACCACTCCCAACAGACC
		Reverse: AAGTGCATCATCGTTGTTCATACA
Mouse	IL-1β	Forward: AAGGGCTGCTTCCAAACCTTTGAC
		Reverse: TGCCTGAAGCTCTTGTTGATGTGC
Mouse	TNF-α	Forward: CATCTTCTCAAAATTCGAGTGACAA
		Reverse: TGGGAGTAGACAAGGTACAACCC
Mouse	GAPDH	Forward: TGAGCAAGAGAGGCCCTATC
		Reverse: AGGCCCCTCCTGTTATTATG

### Western blot analysis

Cells were lysed with cell lysis buffer for 30 min on ice and then centrifuged at 13,000 × *g* for 15 min at 4 °C to clear the lysates. After the debris was pelleted by centrifugation, the supernatants were used for Western blot analysis. The protein concentrations were detected using a BCA protein assay kit (#23225, Thermo Fisher Scientific). Lysate samples comprising 20 or 30 μg of protein were loaded and separated on an 8% to 12% gradient on SDS-PAGE and then transferred onto a polyvinylidene fluoride membrane (Millipore, MA, USA). The membranes were blocked with 5% BSA (BD Bioscience, CA, USA) in TBST (Tris-buffered saline with Tween) buffer and incubated overnight at 4 °C with primary antibodies to VEGF (#SC7269, 1:1,000; Santa Cruz, CA, USA), GAPDH (#ABL1020, 1:2,000; Abbkine, CA, USA), VEGFR2 (#2479, 1:1,000), p-VEGFR2 (#2478, 1:1,000), Akt (#9272, 1:1,000), p-Akt (#4060, 1:1,000), ERK (#9102, 1:1,000), and p-ERK (#4379, 1:1,000; Cell Signaling Technology, MA, USA) and then with horseradish peroxidase-conjugated secondary antibody (GeneTex, MA, USA) for 1 h at room temperature. Electrochemiluminescence reagents (#DG-WPAL250, DoGenBio) were used to detect protein bands. The proteins were then visualized using a Fusion FX6.0 (Vilber Lourmat, Collégien, France) and images were analyzed with Image J software (version 2.9). GAPDH was used as a loading control.

### Wound healing assay

A scratch wound healing assay was used to evaluate cell migration. Briefly, HUVECs were seeded at 10^5^ cells/well in a 12-well plate. After allowing cells to settle for 24 h in culture, the cells in each well were scratched using pipette tips. Following scratching, the cells were washed with PBS, treated under various conditions, and incubated at 37 °C and 5% CO_2_ for 48 h. The phase contrast images were taken by EVOS XL Core (Thermo Fisher Scientific). The wound healing area and the percentage of wound healing were quantified by ImageJ software (version 2.9).

### Tube formation assay

A tube formation assay used HUVECs seeded into culture plates precoated with Cultrex Reduced Growth Factor Basement Membrane Extract (BME; R&D Systems, MN, USA) that was added to a 96-well plate at a volume of 50 μl/well and allowed to polymerize for 30 min at 37 °C. After polymerization, HUVECs were seeded onto BME at 1.5 × 10^4^ cells/well in 100 μl of medium with or without reagents. The cells were incubated at 37 °C in a 5% CO_2_ incubator. The tube formation was observed under an inverted microscope after 24 h. Images were captured with EVOS XL Core (Thermo Fisher Scientific). The images were analyzed for the number of nodes, number of junctions, and total sprout length, and the quantification was performed using the “Angiogenesis analyzer” plug-in [32] in ImageJ software (version 2.9).

### Ex vivo aorta ring sprouting assay

All animal experiments were performed under the guidelines of the Institutional Animal Care and Use Committee of Seoul National University (approval number: SNU-230809-2). Briefly, an incision of the abdominal skin of 6-week-old C57BL/6 mice was performed to expose the thoracic cavity and the heart, lungs, and esophagus. The aorta attached to the spine was then excised using scissors and forceps and transferred to a petri dish and all surrounding adipose tissue was removed. The aorta was sectioned into pieces of 1 mm width and embedded in Cultrex Reduced Growth Factor Basement Membrane Extract (BME; R&D Systems, MN, USA) in a ring shape for ex vivo culture. After incubation in Endothelial Cell Growth Medium MV 2 (Promocell) for 7 days to synchronize the time and the level of sprouting, the aortic rings were treated with different conditions for another 7 days. Images were captured using EVOS XL Core (Thermo Fisher Scientific) and the degree of sprouting was quantified by calculating the radial distance of sprouts. To calculate the radial distance of sprouts, phase contrast images were first processed to enhance contrast by removing background noise in ImageJ (version 2.9). Threshold adjustments were used to highlight the sprouts while excluding the aortic ring and empty areas. Circles were drawn around the sprouts and the aortic ring, and the radial distance was calculated by subtracting the radius of the inner circle from that of the outer circle [33]. The number of samples per group was at least 20 to minimize the error of individuals in each group.

### Statistical analysis

All analyses were performed using GraphPad Prism 5 software. Data were analyzed using analysis of variance (ANOVA) and presented as the mean ± SD.

## Results

### Structure prediction of the Ara27 peptide and characterization of Ara27–siVEGF complexes

**Fig. 1.**
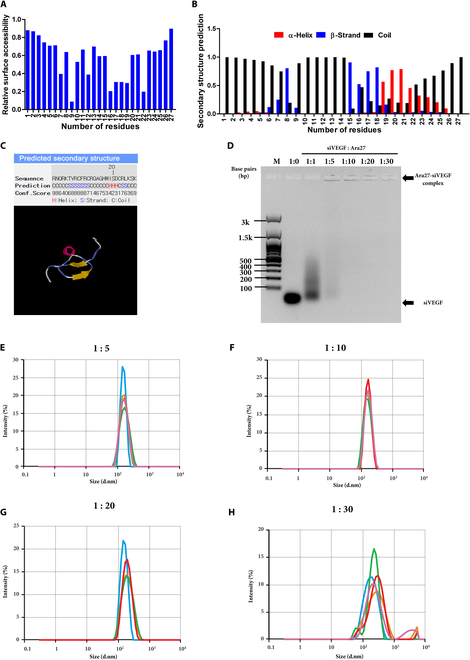
(A) Relative surface accessibility prediction of the Ara27 peptide using the NetSurfP web server. (B) Secondary structure prediction of the Ara27 peptide from the NetSurfP web server. (C) Structural model of the Ara27 peptide predicted by the I-TASSER server. (D) Electrophoretic mobility shift assay to determine optimal molar ratios for Ara27–siVEGF complex formation. A 21-bp siVEGF dsRNA was mixed with Ara27 at molar ratios of 1:1, 1:5, 1:10, 1:20, and 1:30. The brightness and contrast of the image were adjusted for clarity. (E to H) Size distribution of Ara27–siVEGF complexes at various charge ratios using Zetasizer. The molar ratios of siVEGF:Ara27 were (E) 1:5, (F) 1:10, (G) 1:20, and (H) 1:30 with a final siVEGF concentration of 50 nM in all experiments.

The relative surface accessibility (Fig. 1A) and secondary structure prediction (Fig. 1B) of the Ara27, an amphipathic CPP, were analyzed using the NetSurfP web server. The structure predicted by the I-TASSER server, which includes both beta strands and alpha helices (Fig. 1C), was partially consistent with the structure predicted by the NetSurfP web server.

To further investigate the potential of Ara27 for siVEGF delivery to HUVECs, we first determined an optimal ratio of Ara27 complexed with siVEGF by titration at various molar ratios. A mobility shift assay was performed on agarose gel electrophoresis to determine the formation and optimal ratio of Ara27–siVEGF complexes. As shown, molar ratios of siVEGF:Ara27 of 1:5 to 1:30 showed evidence of siVEGF complexes that aggregated in the wells (arrow in Fig. 1D pointing to signals in the respective wells), while this was not evidence with siVEGF alone or a 1:1 mixture with Ara27 where, in both cases, siVEGF was visible as a signal that is less than 100 bp in size. Next, we determined the molecular sizes of the 1:5, 1:10, 20, and 30 complexes using a Zetasizer and found that their mean sizes were 175.5, 158.6, 179.5, and 233.7 nm for 1:5, 1:10, 1:20, and 1:30, respectively (Fig. 1E to H and Table 3).

**Table 3.** Size and polydispersity index of Ara27–siVEGF complex

siVEGF:Ara27 (molar ratio)	Mean complex size (nm)	Polydispersity index
1:5	175.5	0.2119
1:10	158.6	0.0630
1:20	179.5	0.1425
1:30	233.7	0.3536

### Cytotoxicity and intracellular uptake of the CPP–siVEGF complex

**Fig. 2.**
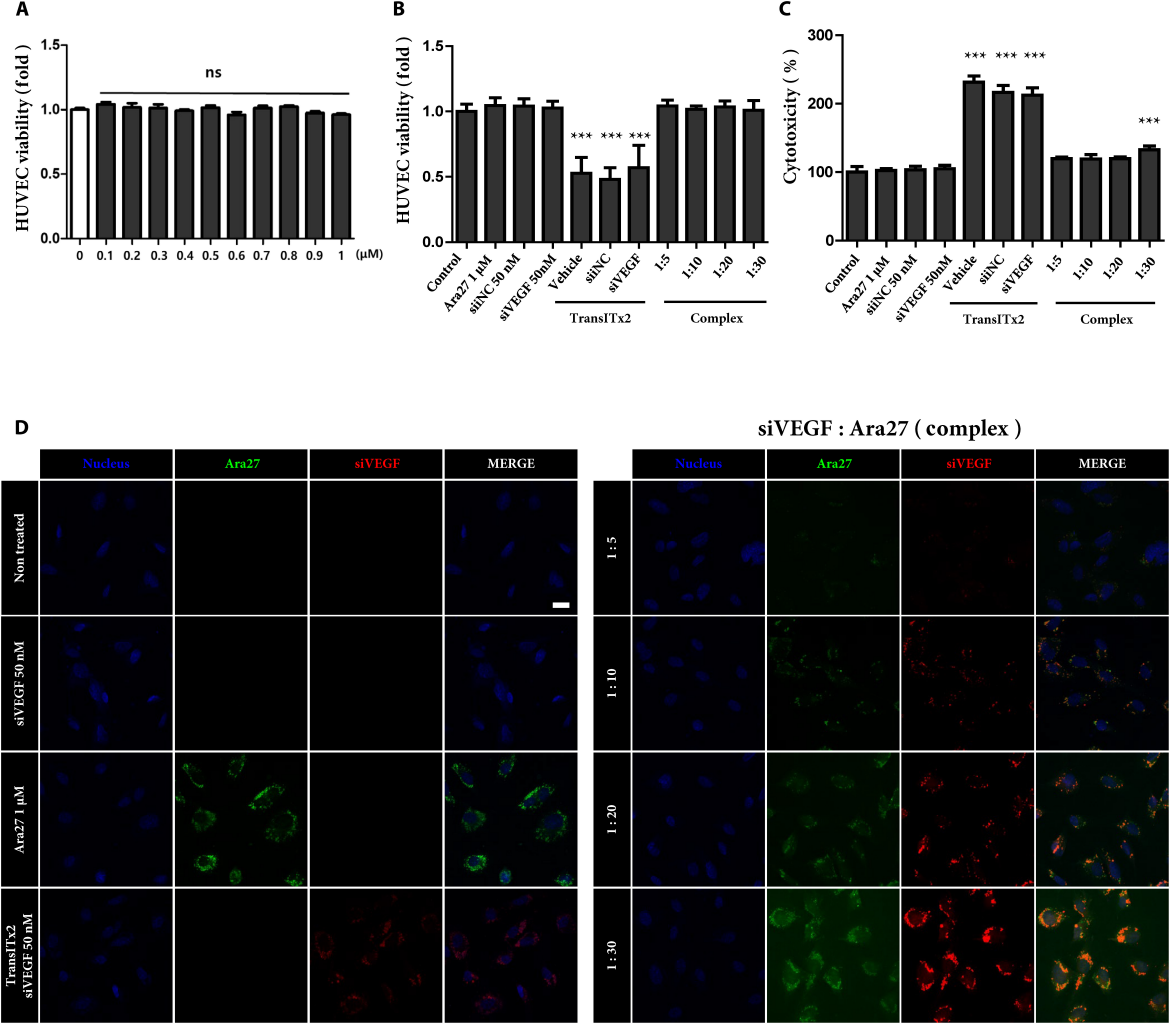
(A) Viability of HUVECs according to the concentration of Ara27 for 24 h treatment. (B) Viability of HUVECs under various treatment conditions after 24 h (*n* = 3, ***P < 0.001). Treatments included Ara27 at 1 μM, and siNC and siVEGF at 50 nM, with molar ratios of siVEGF:Ara27 of 1:5, 1:10, 1:20, and 1:30, all at a final siVEGF concentration of 50 nM. (C) Cell cytotoxicity measured by LDH after 24-h treatment of HUVECs with various conditions. Treatments included Ara27 at 1 μM, and siNC and siVEGF at 50 nM, with molar ratios of siVEGF:Ara27 of 1:5, 1:10, 1:20, and 1:30, all at a final siVEGF concentration of 50 nM (*n* = 3, ***P < 0.001). (D) Efficiency of cellular uptake of siVEGF in HUVECs assessed by confocal microscopy after 24-h treatment with different conditions. The molar ratios of siVEGF:Ara27 were 1:5, 1:10, 1:20, and 1:30, with a final siVEGF concentration of 50 nM. Nuclei were counterstained with DAPI for confocal laser scanning microscopy analysis (scale bar: 20 μm).

To determine whether the complex induced toxicity in HUVECs prior to transfection, a WST assay was performed under various treatment conditions, as follows. First, Ara27, the amphipathic CPP used for complex formation, was exposed to HUVECs and found not to be cytotoxic, even at a concentration of 1 μM following 24-h incubation (Fig. 2A). As a positive control for this experiment, treatment with TransITx2, a commercial polymeric transfection reagent widely used as a positive control for transfection, showed decreased viability after a single treatment while, in contrast, treatment with siNC and siVEGF alone did not result in detectable viability issues. Furthermore, the viability of HUVECs transfected with Ara27–siVEGF complexes with various ratios was greater than 90% (Fig. 2B). Subsequently, the released LDH was measured to assess relative cytotoxicity in HUVECs. Consistent with the WST results, TransITx2 and siRNA mixture with TransITx2 resulted in a significant increase in LDH, which indicate cytotoxicity. The Ara27–siVEGF complexes at a ratio of 1:30 also induced cytotoxicity, whereas no detectable cytotoxic effects were observed at a ratio of 1:20 or lower (Fig. 2C).

Confocal microscopy was used to monitor the cellular uptake of Ara27–siVEGF complexes with various molar ratios in HUVECs after treatment for 24 h (Fig. 2D). There are 3 images representing a red fluorescence of Cy3-labeled siVEGF, FITC-labeled Ara27 with green fluorescence, and a blue fluorescence of the nucleus. As shown, the intracellular localization of siVEGF was observed in both TransITx2 and Ara27–siVEGF complexes in HUVECs. Compared to TransITx2, the fluorescence of siVEGF was lower at a ratio of 1:5, similar at 1:10, but markedly increased at higher ratios (1:20 and 1:30). However, aggregation of Ara27–siVEGF complexes was observed at 1:30.

These results indicate that Ara27–siVEGF complexes successfully delivered siVEGF into the cytosol of HUVECs, with delivery efficiency increasing alongside the Ara27 dose. Furthermore, overlapping fluorescence in the merged image confirmed the colocalization of Cy3-labeled siVEGF and FITC-labeled Ara27.

### In vitro VEGF silencing effect of the CPP–siVEGF complex

**Fig. 3.**
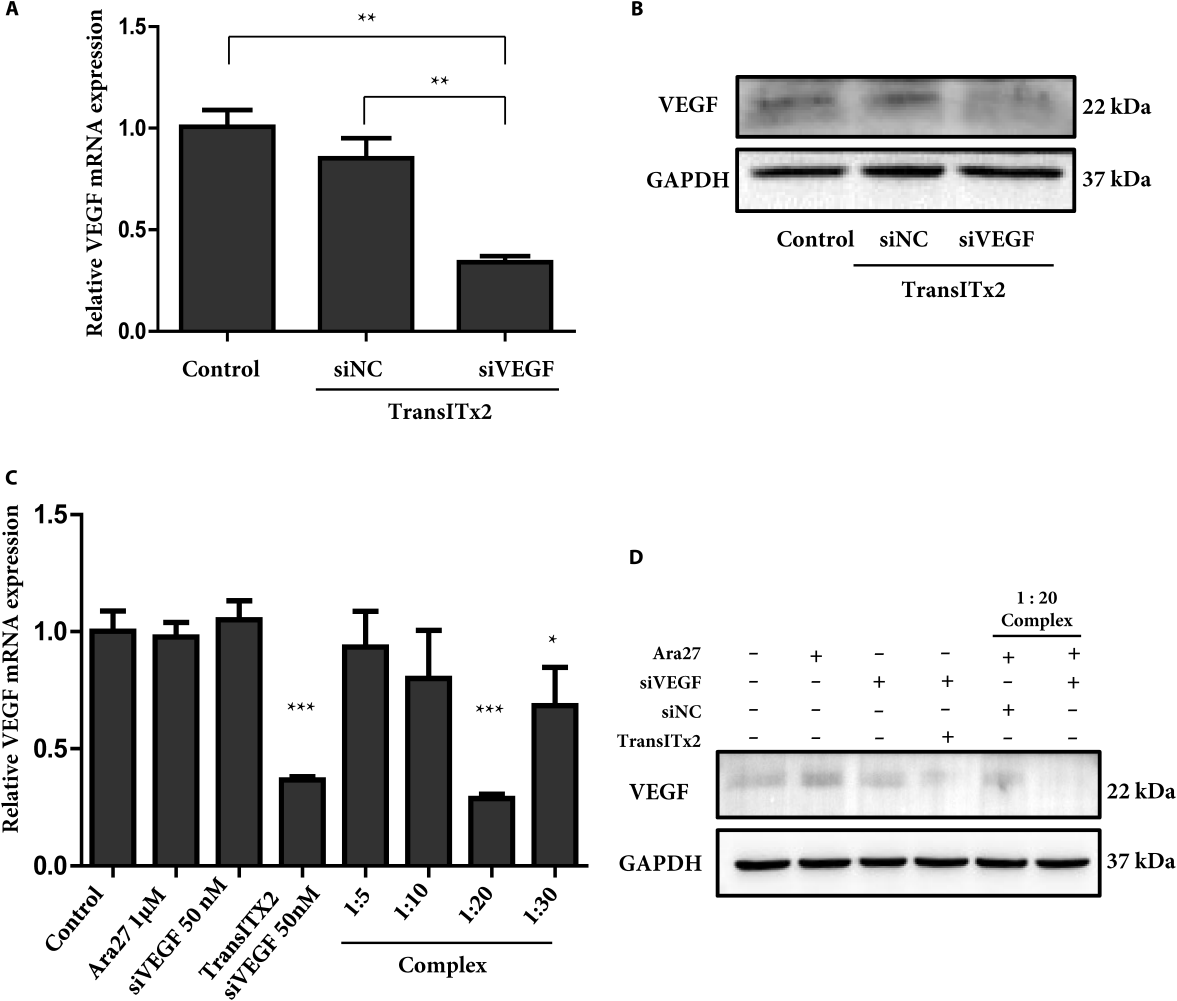
(A) Gene silencing efficiency in HUVECs following siVEGF transfection using TransITx2. The expression levels for VEGF mRNA in cells following treatment for 24 h, assessed by RT-qPCR using GAPDH for normalization. Statistical differences were calculated against control (*n* = 3, **P < 0.01). (B) The expression of VEGF protein expression after 48-h treatment was examined by Western blotting with siNC and siVEGF at 50 nM. (C) Gene silencing efficiency in HUVECs following siVEGF transfection using the Ara27–siVEGF complex. VEGF mRNA expression after treatment for 24 h was assessed, with molar ratios of siVEGF:Ara27 being 1:5, 1:10, 1:20, and 1:30, all at a final siVEGF concentration of 50 nM. Steady-state VEGF mRNA expression levels in HUVECs were assessed by RT-qPCR, with GAPDH signals used for normalization. Statistical differences were calculated against control. (D) The VEGF protein expression levels after 48-h treatment in HUVECs were examined by Western blotting. GAPDH was used as a housekeeping control. Treatments included Ara27 (1 μM), siVEGF (50 nM), TransITx2 + siVEGF (50 nM), Ara27 (1 μM) + siNC (50 nM), and Ara27 (1 μM) + siVEGF (50 nM) complexes, respectively (*n* = 3, ***P < 0.001).

As a positive control, HUVECs were transfected with siVEGF using TransITx2 reagent before the CPP–siVEGF complex was used to assess the knockdown effect of VEGF. An RT-qPCR was used to analyze the expression of VEGF mRNA in HUVECs, and Western blot was used to assess the expression of VEGF protein in HUVECs. Compared to nontreated and siNC-transfected HUVECs by transfection reagent, siVEGF-transfected HUVECs showed effective down-regulation of VEGF mRNA and protein levels (Fig. 3A and B). Next, the CPP–siVEGF complex at various molar ratios was analyzed for its ability to mediate knockdown of VEGF mRNA expression in HUVECs using RT-qPCR. As shown, VEGF mRNA expression was 35% with TransITx2, 90% with the 1:5 complex, 80% with the 1:10 complex, 30% with the 1:20 complex, and 70% with the 1:30 complex. The 1:20 complex clearly reduced VEGF mRNA expression to approximately 30%, which is reminiscent of the capacity for siVEGF knockdown in cells transfected with siVEGF using TransITx2 reagent **(**Fig. 3C). Moreover, VEGF protein expression was also markedly down-regulated by siVEGF delivery when delivered as a 1:20 Ara27–siVEGF complex (Fig. 3D).

These results indicate that the Ara27–siVEGF complex at a 1:20 ratio is optimal for HUVECs, facilitating effective intracellular delivery of siVEGF without inducing toxicity, while also effectively suppressing both VEGF mRNA and protein expression.

### Inhibition of angiogenesis by the Ara27–siVEGF complex is associated with changes to VEGF/VEGFR2-mediated Akt and ERK signaling pathways

**Fig. 4.**
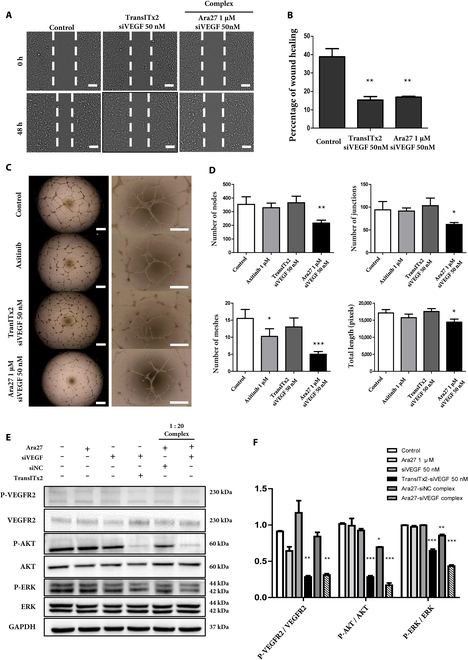
(A) Wound healing assay and quantitative analysis of wound healing area after 48 h of treatment. Representative migration images of HUVECs at different time points (0 and 48 h) for different groups (scale bar: 50 μm). (B) Quantitative analysis of migration ability by calculating percentage of wound healing area (*n* = 3, **P < 0.01). (C) Effect of Ara27–siVEGF complex on HUVEC tube formation assay. Representative images of HUVECs after 24 h of treatment (scale bar: 50 μm). (D) Quantitative analysis of the total nodes, junctions, meshes, and length using ImageJ (*n* = 3, *P <0.05; **P < 0.01; ***P < 0.001). (E) The protein expression levels of the VEGF/VEGFR2/ERK/Akt signaling pathway after 48-h treatments. GAPDH was used as a housekeeping control. Ara27 (1 μM), siVEGF (50 nM), TranITx2 siVEGF (50 nM), Ara27 (1 μM) + siNC (50 nM), and Ara27 (1 μM) + siVEGF (50 nM) complex, respectively. (F) Quantitative analysis of protein expression. (*n* = 3, *P <0.05; **P < 0.01; ***P < 0.001).

Next, we explored whether treatment of HUVECs with Ara27–siVEGF complexes at 1:20 influenced VEGF expression to affect their cellular behaviors. To test this, we first performed a scratch wound healing assay to evaluate HUVEC migration activity, given the importance of endothelial cell mobility during angiogenesis. As shown, cell migration was inhibited when HUVECs were treated with TransITx2 and siVEGF, or with Ara27–siVEGF complexes. The percentage of HUVEC migration was reduced to less than 20% in both of these groups, while the migration of nontreated HUVEC was about 40% after 48-h incubation (Fig. 4A and B). Next, we tested the effects of Ara27–siVEGF on the ability for HUVECs to form tube-like structures in vitro. As a positive control, HUVECs treated with axitinib, an inhibitor of the VEGF receptor, were used. As shown, compared to the control groups, HUVECs treated with Ara27–siVEGF exhibited less complex tube networks with a fewer number of nodes, junctions, and meshes (Fig. 4C and D).

VEGFR2 is a major player in VEGF-mediated angiogenesis [34]. Several downstream protein kinase pathways such as the RAF/MEK/ERK and PI3K/Akt pathways are closely involved in endothelial cell proliferation, cell survival, and migration that can modulate angiogenesis. As such, we wanted to quantify potential changes in the steady-state levels for these proteins using Western blot as a measure of the impact on signaling through such pathways as a consequence of Ara27–siVEGF treatment (Fig. 4E and F). As shown, we found decreased immunoblotted signals for p-VEGFR2, p-Akt, and p-ERK in HUVECs transfected with both TransITx2 and siVEGF, as well as in HUVECs treated with Ara27–siVEGF complexes. Therefore, these results indicate that a VEGF/VEGFR2/Akt/ERK signaling pathway is associated with the inhibition of angiogenic-like behaviors, such as cell migration and the formation of tube-like structures by HUVECs in culture.

### Inhibition of angiogenesis revealed in an ex vivo aorta ring assay following treatment with Ara27–siVEGF complexes

**Fig. 5.**
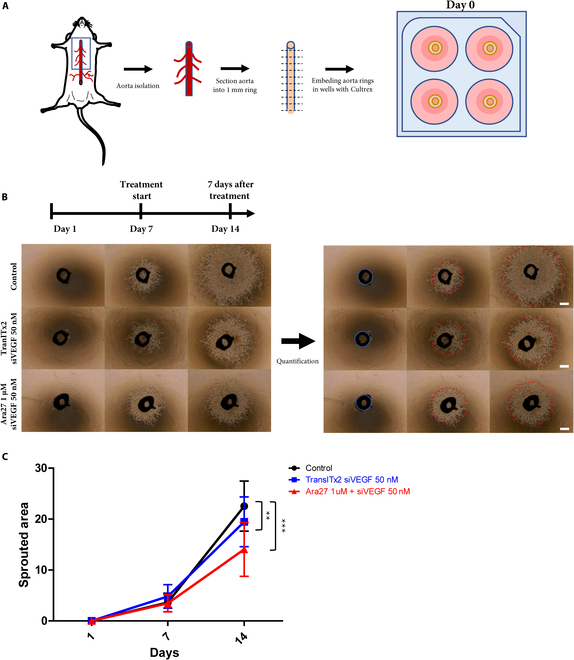
(A) Schematic illustration of ex vivo mouse aorta ring sprouting assay. (B) Representative images of the aortic ring assay under various treatment conditions (TranITx2 + siVEGF [50 nM], and Ara27 [1 μM] + siVEGF [50 nM] complexes), respectively (scale bar: 50 μm). (C) Quantitative analysis of sprouted area (**P < 0.01; ***P < 0.001). The number of samples in each group exceeded 20.

The inhibitory effect of Ara27–siVEGF complexes at 1:20 was investigated in an ex vivo mouse aortic assay as a way to support the in vitro cell studies. Briefly, aorta tissues were isolated from C57BL/6 mice, dissected into 1-mm-width rings and the aorta rings were embedded and cultured in Cultrex, which serves as an extracellular membrane environment (Fig. 5A). The aortic rings were cultured in HUVEC growth medium for 7 days to synchronize the time point and level of sprouting and then treated in several groups for another 7 days. As shown, compared to the control group, both the tissues treated with TransITx2 and siVEGF, as well as the Ara27–siVEGF treatment groups showed distinct reductions in the quantified sprouted areas for the aorta rings on day 14 (Fig. 5B). The number of individuals in each group exceeded 20. Quantification of the sprouted areas revealed that samples treated with Ara27–siVEGF complexes exhibited the most significant reductions in sprouting (Fig. 5C). These studies demonstrate that treatment with Ara27–siVEGF complexes inhibited endothelial cell proliferation, which reduced sprouting from the aortic ring and consequently inhibited angiogenesis.

### Examining the immunotoxicity of Ara27–siVEGF complexes in various macrophage cell lines

To evaluate the effect of Ara27–siVEGF complexes on immunotoxicity, human monocyte THP-1 cells and mouse macrophage RAW 264.7 cells were used.

**Fig. 6.**
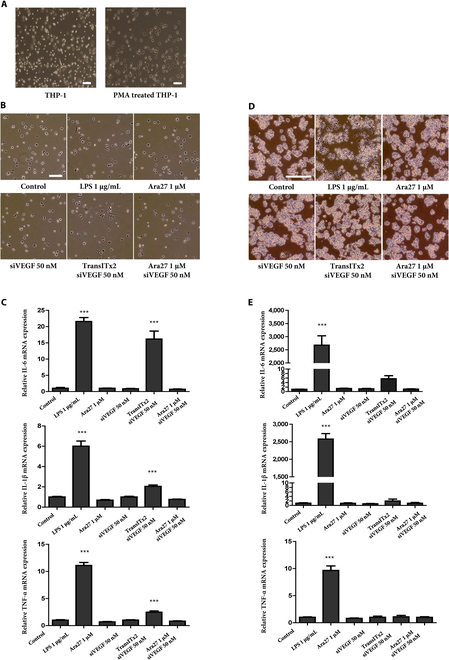
(A) Morphology of THP-1 and THP-1-derived macrophages (scale bar: 20 μm). (B) Morphological changes of THP-1-derived macrophages after various treatments for 24 h (scale bar: 20 μm). (C) Pro-inflammatory cytokine mRNA expression (IL-6, IL-1β, and TNF-α) in THP-1-derived macrophages following treatments for 24 h (***P < 0.001) (scale bar: 20 μm). (D) Morphological changes of RAW 264.7 cells after various treatments for 24 h. (E) Pro-inflammatory cytokine mRNA expression (IL-6, IL-1β, and TNF-α) in RAW 264.7 cells after various treatments for 24 h (***P < 0.001).

THP-1 cells were first differentiated into macrophages by treatment with PMA for 48 h. The morphology of THP-1-derived macrophages, which can be distinguished from THP-1 monocytes by their adherence to culture plates, was confirmed (Fig. 6A). After treatment with 1 μg/ml LPS and TransITx2-mediated transfection, THP-1-derived macrophages showed a change to a linear morphology after 24 h, while other groups showed no morphological changes (Fig. 6B). Correspondingly, the mRNA expression levels of the pro-inflammatory cytokines interleukin-6 (IL-6), IL-1β, and tumor necrosis factor-α (TNF-α) were markedly up-regulated by LPS treatment and significantly increased after TransITx2 transfection (Fig. 6C). In RAW 264.7 cells, substantial morphological changes and increased mRNA expression levels of IL-6, IL-1β, and TNF-α were observed upon LPS treatment alone (Fig. 6D and E).

Overall, the results showed that the Ara27–siVEGF complex did not induce immunotoxicity in several macrophage cell lines, whereas TransITx2 posed a potential risk of immunotoxicity.

## Discussion

The CPP used in this study, Ara27, was found to effectively form complexes with siVEGF through charge-binding interactions. It has been shown that Ara27 can be incorporated into various cell lines at low concentrations under short treatment conditions without cytotoxicity [26]. A significant improvement in intracellular uptake was seen with Ara27 compared to commonly used CPPs, such as Tat-protein transduction domain and membrane translocating sequence, without adverse effects on cell viability [27].

The molar ratio for CPPs in complex with siRNAs has an effect on the formation of stable CPP–siRNA complexes. A previous study showed that siRNAs, when complexed with amphipathic CPPs in molar ratios above 1:15, achieved superior results in gene silencing [35]. In the present study, we found that molar ratios of siVEGF to Ara27 at a range between 1:10 and 1:30 were optimal to form complexes.

The size of the complex is also an important factor when determining the capacity for CPP–siRNA intracellular uptake, and to overcome potential risk of cellular toxicity. Previous studies indicated that CPP–siRNA complexes of less than 200 nm were desirable [36,37]. Our DLS analysis showed that the average size and size distribution of the Ara27–siVEGF complexes (that is, the PDI) with a molar ratio of 1:5, 1:10, and 1:20 were less than 200 nm. However, the complexes from a ratio of 1:30 were found to have a size distribution larger than 200 nm, with a large fluctuation in the values. Notably, CPP–siRNA PDI values greater than 0.7 are indicative of a very broad complex size distribution in the sample that may not be suitable to be analyzed by DLS [38]. In contrast, values of 0.2 or below are typically considered acceptable for polymer-based nanoparticles [39]. The PDI value of complexes at a formulation ratio of 1:30 is higher than 0.35, suggesting that the complex size distribution is highly unstable, which could be caused by the aggregation of CPPs. In our study, the complexes from a ratio of 1:30 caused cytotoxicity and aggregation of complexes.

Cationic lipids and polymers are widely used for nucleic acid delivery into cells in vitro and in vivo [40,41]. However, the clinical applicability of many cationic vectors developed so far has been restricted by their substantial toxicity at working concentrations. For these, a relationship between charge and cellular processes is evident, as excess positive charges on the complex surface can interact with cell membranes to inhibit normal cellular functions and cell survival signaling [42]. For in vivo applications, cationic lipids are often the cause of acute inflammatory responses. On the other hand, cationic polymers, such as polyethyleneimine, induce cell necrosis, apoptosis, and autophagy [43].

HUVECs are recognized to be difficult to transfect and vulnerable to the toxic effects of transfection reagents [44]. The commercial polymeric transfection reagent, TransITx2, was used as a positive control for in vitro transfection efficiency. Cell viability decreased significantly despite treatment with transfection reagent alone, while Ara27 maintained viability above 90% and no cytotoxicity was caused in both Ara27 alone and multiple ratios of complex treatments (1:1, 1:5, 1:10, and 1:20). Also, the complex at a 1:20 ratio demonstrated the highest VEGF knockdown efficiency without any concerns regarding toxicity in HUVECs. These results indicate that Ara27 might be suitable for siRNA delivery in vitro without cytotoxicity concerns, but additional tests with more measures of cytotoxicity will strengthen this finding in our current study.

Based on our evaluation of the ratio, size distribution, homogeneity of the complexes, cell viability, cytotoxicity, and VEGF knockdown efficiency, we conclude that a siVEGF:Ara27 ratio of 1:20 leads to the formation of the most appropriate complexes to efficiently deliver siVEGF to HUVECs in vitro.

Endothelial cell migration is essential to angiogenesis and is mediated by VEGF signaling [45]. Here, we found that HUVEC migration was suppressed by treatment with Ara27–siVEGF complexes. This might be associated with cytoskeletal function, which is essential for modulating cell motility. Furthermore, in the process of angiogenesis, tube formation is highly dependent on the migration of endothelial cells [46], and we found that treatment with Ara27–siVEGF disrupted tube formation as well. As a major signaling pathway in angiogenesis and cell survival, VEGF binds to VEGFR2, phosphorylating and triggering a cascade of signaling that contributes to angiogenesis, permeability, or survival [34]. Numerous VEGFR2 downstream signaling mediators, such as Akt and ERK, have also been found to regulate endothelial cell survival and proliferation [47]. In our experiments, we found that treatment with Ara27–siVEGF complexes inhibited angiogenesis and led to a decrease in phosphorylation of VEGFR2, Akt, and ERK, but not the steady-state levels of each of these proteins in HUVECs.

Then, treatment with Ara27–siVEGF suppresses sprouting in an ex vivo aorta ring assay, a model for angiogenesis. The use of ex vivo models of angiogenesis has gained prominence, particularly in the context of overcoming the limitations of in vitro techniques and simplifying the complexity of in vivo models. When aorta rings were embedded in extracellular matrix such as Cultrex, the endothelial cells that line the aortic intima are induced to migrate and form 3-dimensional capillary-like structures [33]. That Ara27–siVEGF treatment disrupts the formation of these structures offers strong evidence for VEGF knockdown in these tissue preparations.

The immunotoxicity of biomaterials is vital for developing safe and effective therapeutic applications, as their interaction with the immune system can significantly influence both efficacy and safety [48,49]. Therefore, studying how these materials interact with various immune cells is essential to understand the resulting immunological responses in therapy. In our study, the Ara27–siVEGF complex did not induce immunotoxicity in either THP-1-derived macrophages or RAW 264.7 cells, while TransITx2 exhibited a potential risk of immunotoxicity.

**Fig. 7.**
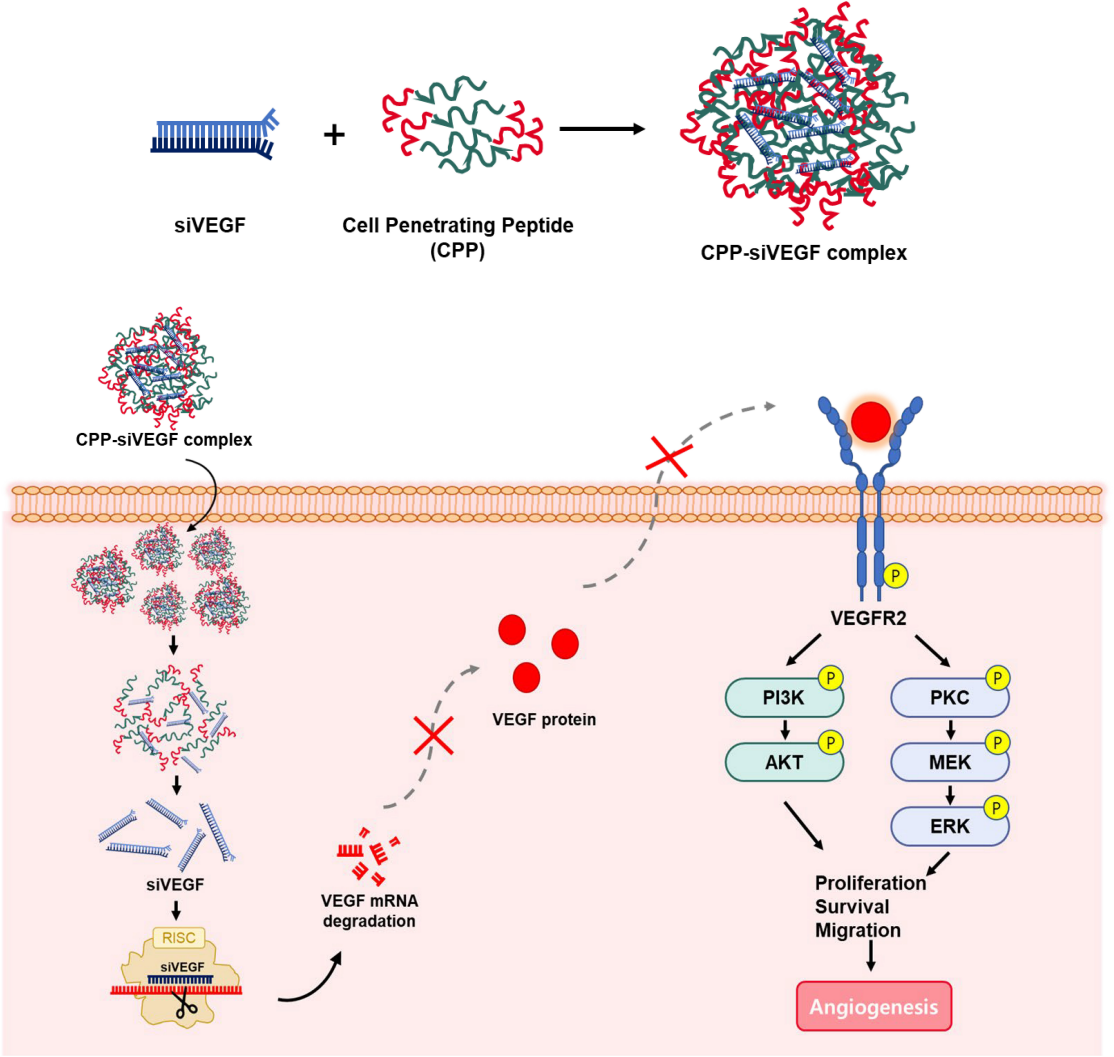
A schematic illustration of the putative mechanism for angiogenesis in HUVECs by treatment with Ara27–siVEGF complexes.

In conclusion, we demonstrate that the Ara27–siVEGF complex is a promising candidate for delivering siVEGF. Prepared in an optimal 1:20 ratio, this complex efficiently delivered siVEGF to HUVECs, resulting in decreased VEGF mRNA and protein expression without inducing cytotoxicity or aggregation. In addition, the Ara27–siVEGF complex inhibited angiogenesis by suppressing VEGFR2-mediated Akt and ERK phosphorylation (Fig. 7). Treatment with Ara27–siVEGF also suppressed endothelial cell migration in a scratch wound assay and impaired their capacity to form vascular networks while disrupting efficient signaling through the VEGFR2-dependent Akt and ERK pathways. These in vitro effects of Ara27–siVEGF on angiogenesis in HUVECs were further supported by findings from an ex vivo mouse aorta ring sprouting assay, which showed that treatment with Ara27–siVEGF suppressed sprouting. Notably, the Ara27–siVEGF complex did not induce immunotoxicity in either THP-1-derived macrophages or RAW 264.7 cells, while TransITx2 exhibited a potential risk of immunotoxicity. Overall, these results suggest that Ara27 is a potentially effective approach for siRNA delivery to modulate angiogenesis while maintaining an immunological profile.

## Data Availability

The datasets used and/or analyzed during the current study are available from the corresponding authors on reasonable request.
