# Supplementary material for: A Novel Cell-Penetrating Peptide–Vascular Endothelial Growth Factor Small Interfering Ribonucleic Acid Complex That Mediates the Inhibition of Angiogenesis by Human Umbilical Vein Endothelial Cells and in an Ex Vivo Mouse Aorta Ring Model
Source: Biomater Res. 2025 Jan 7;29:0120. doi: 10.34133/bmr.0120 (PMC11704089; doi:10.34133/bmr.0120)
Supplement: Supplementary 1 — Table S1 [file bmr.0120.f1.docx]

**Supplementary Materials**

**Table S1**. Fluorescence information used in confocal microscopy

| **Fluorescence** | **DAPI** | **FITC** | **Cy3** |
| --- | --- | --- | --- |
| **Channel Color** | Blue | Green | Red |
| **Excitation Wavelength (nm)** | 353 | 495 | 548 |
| **Emission Wavelength (nm)** | 465 | 519 | 561 |
| **Detector Gain (V)** | 640 | 580 | 790 |
| **Detector Digital Gain** | 1.0 | 1.0 | 1.0 |
